# 肺癌术中并发脑梗塞1例报告

**DOI:** 10.3779/j.issn.1009-3419.2010.05.34

**Published:** 2010-05-20

**Authors:** 梅岭 李, 洪芹 何, 文进 王, 健莉 王

**Affiliations:** 061000 沧州，河北省沧州市人民医院肿瘤科二区 Department of Medical Oncology, the People's Hospital of Cangzhou, Cangzhou 061000, China

目前，临床上常采用肺叶切除或全肺切除术治疗肺癌，但术中会发生瘤栓弥散至大脑形成脑组织大面积、弥散性栓塞，可给患者带来不可逆的、永久的、致命的并发症，预后恶劣，在临床上少见。我院肿瘤科在临床上遇到1例，在此，特报道如下。

## 1 临床资料

患者，男性，38岁。主因干咳伴右胸背疼痛20天入院。患者于20天前，无明显诱因地出现干咳，无痰，偶有少许白色粘痰不易咳出，无咳血。同时伴有右胸背疼痛。无其它不适。当地医院胸透发现右肺占位后，未行进一步诊治而来我院治疗。既往体健。入院查体：T 36.5 ℃，P 86次/分，R 19次/分，BP 120 mmHg/75 mmHg。神清合作，气管居中，胸廓无畸形，呼吸动度一致，右下肺语颤略增强，双上肺叩清音，右下肺叩实音，肺肝浊音界不清，两上肺呼吸音清晰，右下肺呼吸音微弱，未闻及干湿性罗音；心脏未触及震颤，未闻及病理性杂音，腹平坦，无胃肠型及蠕动波，全腹无压痛，未触及肿块，全腹叩鼓音，肠鸣音正常。四肢活动正常。辅助检查：血常规、尿常规、便常规、肝肾功能等均未见异常。胸大片示右肺中下叶占位，考虑为右侧中心型肺癌。胸部CT提示右肺中下叶巨大肿物并肺不张，侵犯心包，考虑为右侧中心型肺癌，建议穿刺活检（图 1）。经皮肺穿活检病理报告考虑良性病变的可能性大，但不能彻底除外恶性。脑CT未见异常。未行心脏超声及造影检查。入院诊断：右肺中下叶巨大肿物并肺不张，右侧中心型肺癌不除外。经积极的术前准备，择期在全麻下行开胸探查术。术中发现巨大肿物，约20 cm×15 cm×10 cm，位于右肺中下叶，已侵犯上叶和心包，肿瘤包绕右肺门血管，特别是肺门有多枚淋巴结肿大，崁顿与右肺门部肺动、静脉及右支气管处，且有融合现象。但未发现肺静脉内有癌栓。速冻活检病理报告右肺腺鳞混合癌。决定行心包内处理肺血管右全肺切除术。小心地解剖，暴露右肺门，清除多枚淋巴结，打开心包，小心地游离右肺动脉并双重结扎和缝扎，离断右肺动脉；小心地游离右肺静脉并双重结扎和缝扎，离断右肺静脉；进一步处理右肺支气管，离断并缝扎。移除标本送检。手术过程顺利。术后患者不能如期醒来，且发现患者高热，T 39.3 ℃，P 136次/分，规整，BP 120 mmHg/70 mmHg。双侧瞳孔扩大，右侧直径4.5 mm，左侧直径4.5 mm，对光反射迟钝。但呼吸平稳，R 24次/分，指端脉搏氧97%-99%。立即行头颅CT扫描，发现脑组织大面积、弥散性栓塞。考虑术中瘤栓弥散至大脑（图 1）。立即转ICU重症监护病房进行抢救性治疗。术后4天拔除胸腔引流管，10天伤口拆线。至术后17天，患者仍不能清醒，家属放弃治疗自动出院。术后20天死亡。术后病理报告：肉眼观：标本为右肺上、中、下叶，肿物位于右肺中下叶近肺门处，侵犯上叶，大小为：18.5 cm×16 cm×13.8 cm。未发现支气管及肺动、静脉断端有瘤破坏或栓样物。镜下检查提示：右肺上、中、下叶低分化鳞癌，侵犯脉管，可见脉管内瘤栓；局部区域有腺癌样分化；支气管断端（-）；纵隔组织（+）；心包组织（-）；淋巴结未见癌转移（0/26）：隆突下淋巴结（0/5），右肺门淋巴结（0/8），肺段淋巴结（0/1），支气管周围淋巴结（0/12）；肺门血管受累，肺动脉及静脉内可见瘤栓（图 2）。

**1:**
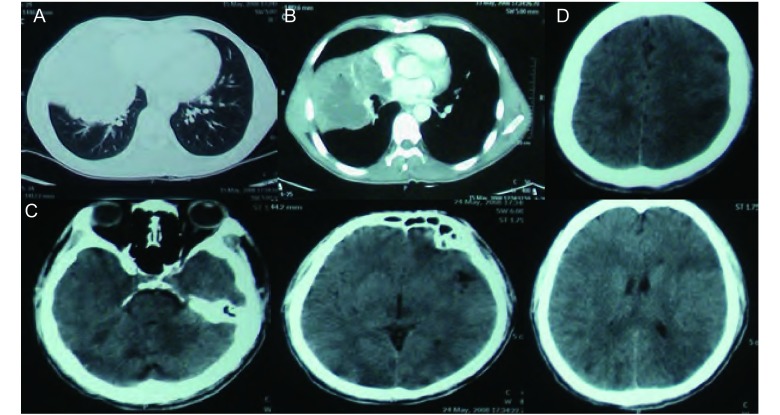
典型的术前肺部CT扫描片和典型的术后头部CT扫描片。A：肺窗显示右肺中下叶肺门处巨大肿物；B：纵隔窗显示右肺中下叶巨大团块状软组织影，侵犯心包；C：显示阻塞的基底动脉末端引起基底动脉尖综合征；D：显示左侧大脑中动脉分支闭塞引起顶叶脑栓塞 The classical CT scan of lung before operation and cephalosome after operation. A: Chest CT scan of lung shows huge tumor of the right lung; B: Chest CT scan of mediastinum shows huge conglomeration of parenchyma in the right lung.It encroach pericardium; C: It shows that obstructed basilar artery lead to TOBS; D: It shows cerebral embolism of parietal lobe because the ramus of the left middle cerebra, artery is obturated

**2:**
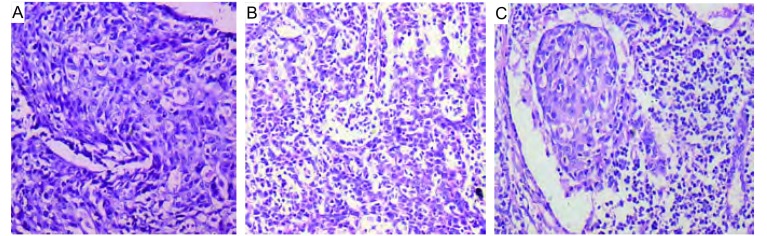
术后病理常规石蜡切片（HE，×400）。A：显示低分化鳞状细胞癌；B：显示鳞状细胞癌伴有腺样分化；C：显示脉管内瘤栓 Pathological paraffin section after operation (HE, ×400). A: It shows pooly differentiated squamous cell carcinoma; B: It shows that squamous cell carcinoma have glandulose differentiation; C: It shows embolism of tumor in the vessel

## 2 讨论

到目前为止，肺癌的早期诊断手段尚不完善，大部分肺癌患者来院诊治已属中、晚期，95%的未经治疗的患者在1年内死亡^[1]^。1933年Graham首次用全肺切除术治疗肺癌成功，至今已76年，由于全肺切除术后并发症较多，所以多年来肺癌的全肺切除术已逐渐减少，但在临床上仍有其应用价值^[2, 3]^。特别是一些晚期肺癌、病变比较广泛的患者，全肺切除术是其减轻或消除肿瘤负荷，为患者的续后综合治疗创造有利条件、延长患者寿命、提高生活质量的有效措施。心包内处理血管切除全肺在扩大肺癌手术范围中的应用价值已为临床充分肯定，其远期预后与心包外标准全肺切除术相近^[4]^，对于那些由于肿瘤侵及肺门大血管或周围重要脏器而呈“冻结”状态、常规肺切除技术难以切除病变，采用心包内处理肺血管行肺切除以及心房部分切除术，可使一部分患者获得手术机会，并且可以使一些过去不能被完全切除的肺癌达到完全性切除，为下一步放、化疗及免疫治疗打下基础^[5]^，所以，临床医生可以大胆地选择。但是，最近我们遇到的这例患者，在术中发生脑组织大面积、弥散性栓塞，虽未能尸检证实，但从分析看，以肺癌癌栓脱落致脑组织大面积、弥散性栓塞的可能性最大。给患者带来不可逆的、永久的、致命的并发症，预后恶劣。这值得我们临床医师注意。

恶性肿瘤在手术过程中，不可避免地会出现瘤栓进入血液循环系统，从而造成手术中的医源性的扩散，这也是肿瘤外科医师注重手术中无瘤原则的根据。复习有关这方面的医学文献发现，因为肺癌手术过程中出现瘤栓性大面积脑栓塞给患者造成不可逆的致命的并发症相关报道很少。林惠民等^[6]^曾报道过1例肺癌术中脑转移并发术后脓胸死亡；李镇维^[7]^报道过1例肺癌术中并发脑梗塞；蓝之源等^[8]^报道过1例因肺癌术中癌栓被夹碎，次日死于急性脑栓塞；梁小平等^[9]^曾报道过1例右肺中叶肺癌术后发现有右上肢血栓（或瘤栓）形成；林链风等^[10]^报道过术后第1天发生脑栓塞、右髂动脉癌栓栓塞最后致右足干性坏疽发生。

肺癌手术中出现瘤栓性大面积脑栓塞，这与术中术者操作过程中挤压肿瘤造成瘤栓脱落有关。其教训是：①术中先行处理肺静脉。有人曾观察肺静脉注入心房血流中可因手术操作而有“阵雨样瘤细胞群”。可见先行处理肺静脉是非常重要的。②术中发现肺静脉内异常，先行切开肺静脉取出瘤栓后再处理肺静脉是正确的。术中行B超检查，证明有肺静脉瘤栓形成后，立即改为心包内肺血管解剖控制切除术^[11]^，以预防术中瘤栓因挤压脱落引起的相关并发症。③如不能先行处理肺静脉而必须先行处理肺动脉时，可向肺动脉内注入抗癌药（如：顺铂），或采取肺门阻断术。④术前反复进行经皮肺穿，取得病理学的确诊后，行新辅助放化疗，使瘤体在一定程度上缩小，然后再行手术。⑤手术在体外循环下进行。肿瘤医学网于2009年1月15日文章《体外循环技术用于晚期肺癌手术》中提到，将体外循环技术用于肺癌的外科治疗是肺癌外科治疗的重要进展。近10余年来，对于局限性进展期肺癌，国内外学者尝试将体外循环技术应用到了肺癌的外科治疗，得到了非常好的治疗效果。其优点是：首先，由于术中心脏和肺均无血流，避免了常规手术方法容易导致的意外性大出血，从而使常规手术方法不能切除的肺癌获得根治性切除；其次，手术无需换气，可将气管、支气管任意开放，从而有利于施行较复杂的气管、支气管和隆凸重建手术；最后一点，它还可以减少术中因剥离压迫肿瘤而导致的肿瘤细胞血行播散的机会。对于肺癌侵及肺静脉根部或肺静脉内有癌栓形成的患者，国外学者早就提倡在体外循环下手术^[12]^。本例患者如术前行心脏超声或心脏造影，可能发现异常，如在体外循环下进行手术，会避免术中瘤栓播散。

臧德安等^[13]^提出肺癌手术无瘤操作技术共30项，值得胸外科医师在临床工作中借鉴。
